# Perspectives on Imaging the Left Main Coronary Artery Using Intravascular Ultrasound and Optical Coherence Tomography

**DOI:** 10.3389/fcvm.2014.00016

**Published:** 2015-01-09

**Authors:** Harry C. Lowe

**Affiliations:** ^1^Concord Repatriation General Hospital, Sydney, NSW, Australia

**Keywords:** coronary angiography, intravascular ultrasound, optical coherence tomography, left main coronary artery, imaging

## Abstract

Percutaneous coronary intervention (PCI) for significant left main coronary artery (LMCA) stenosis is increasingly being viewed as a viable alternative to coronary artery bypass grafting (CABG) (1). This is leading to an expectation of increasing numbers of such procedures with a consequent focus on both the ability to image lesion severity and assess more accurately the results of PCI. While there have been advances in physiological assessment of left main severity using fractional flow reserve (FFR) and in non-invasive assessment of the left main using coronary computerized tomography CT (2), imaging of the LMCA using intravascular ultrasound (IVUS) and more recently optical coherence tomography (OCT) has the specific advantage of being able to provide very detailed anatomical information both pre- and post-PCI, such that it is timely to review briefly the current status of these two imaging technologies in the context of LMCA intervention. This is presented specifically contrasting the use of these technologies both in pre-PCI lesion severity assessment, and peri-PCI procedural evaluation. Not discussed here is the separate issue of longer-term surveillance of asymptomatic patients having undergone LMCA stenting, which may appropriately be performed non-invasively using coronary CT, reviewed in detail elsewhere (2).

## Left Main Coronary Artery Stenosis

The LMCA refers to the proximal coronary segment generally arising from the left coronary sinus and extending to bifurcate into the left anterior descending coronary artery and the circumflex coronary. It is generally considered to comprise three distinct anatomical portions; the ostium, body, and distal portion (3). The LMCA contains a higher elastic tissue component compared to the rest of the coronary tree (4) and supplies large myocardial territory, making significant disease of the LMCA often associated with severe ischemia, arrhythmias, and other life-threatening sequelae.

## PCI for LMCA Stenosis

**Figure 1:**
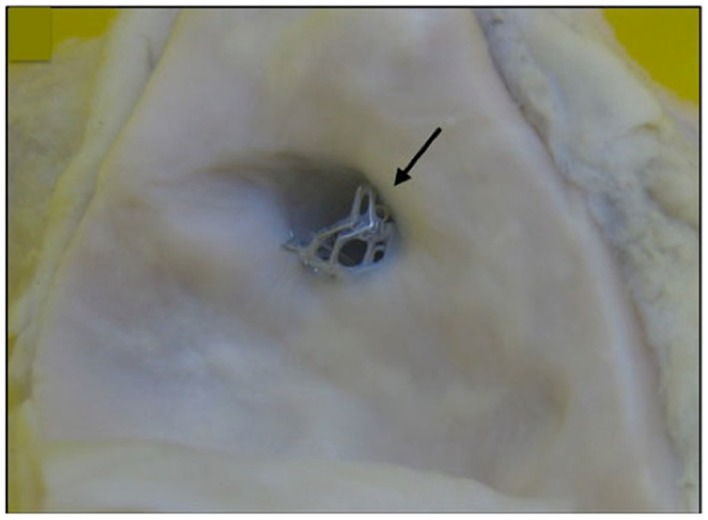
**A bare metal stent (4.0 mm × 13 mm Vision) placed suboptimally in proximal left main coronary artery, extending into aorta taken at post-mortem (marked by arrow)**. There is proximal extension of the stent to within the aorta of 2–3 mm.

Percutaneous coronary intervention for LMCA stenosis has and continues to be evaluated against conventional CABG in selected patients, with favorable results, reviewed elsewhere (1). Event rates are modest, but lower than for CABG in some groups (5 vs. 7.9% at 3 years for combined death and MI: PCI vs. CABG, *p* < 0.001) (1), and mean that further improvements in outcomes may be expected with improvements in imaging and techniques (Figure 1).

## IVUS for LMCA Stenosis

**Figure 2:**
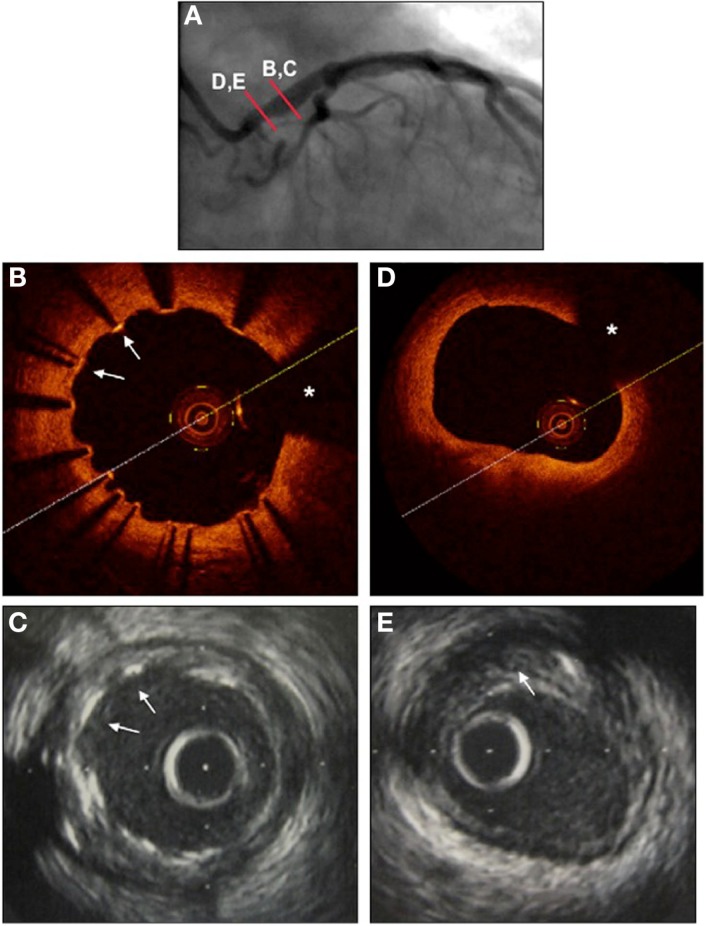
**Left main coronary artery imaged with angiography (A), optical coherence tomography (B,D), and intravascular ultrasound (C,E), 1 year following stenting using a drug-eluting stent**. **(A)** Coronary angiography RAO view. Proximal mild left main lesion and dilated stent in mid and distal portion of left main coronary artery. **(B)** OCT to mid left main. Stent struts are seen well-apposed to the vessel wall, with precise detail (arrows). Wire artifact shadow marked as asterisk. **(C)**. IVUS to mid left main at same site. Stent struts are seen, and appear well-apposed, but without the precision of the OCT images (arrows). **(D)** OCT to ostium of left main. The lumen is very clearly seen (area calculated at 7.1 mm^2^), but no detail is provided as to the nature or extent of underlying atherosclerotic plaque. Wire artifact shadow marked as asterisk. **(E)** IVUS to ostium of left main at same site. The lumen is well visualized (lumenal area calculated at 6 mm^2^). The extent of underlying atherosclerotic plaque is evident, marked with arrow (calculated at 40% area stenosis).

Intravascular ultrasound has been long considered the gold standard imaging modality for the assessment of LMCA stenosis severity, prior to any intervention, and has been elegantly reviewed elsewhere (5). Differences in outcomes have been reliably demonstrated by a number of investigators using IVUS defined binary cut-offs for lesion severity, with minimum lumen areas (MLA) of between 6 and 7.5 mm^2^ as defining significant LMCA stenosis severity (6, 7). Improved outcomes have been documented in those patients with severe LMCA stenosis lesion undergoing revascularization compared to those treated medically (5). Interestingly, while IVUS is uniquely able to assess plaque volume in this context (Figure 2), such estimates of plaque volume have not been used clinically to add precision to assessments of MLA.

Intravascular ultrasound has also been used to assess stent strut apposition and peri-procedural complications post-PCI, although IVUS image quality of stent strut apposition in particular does have limitations (Figure 2). Non-randomized registry data from 975 patients undergoing PCI vs. CABG for LMCA stenosis suggested improved outcomes with IVUS guided PCI compared to angiography alone (mortality 4.7 vs. 16% at 3 years, *p* = 0.048) (8). These and other data have supported the general clinical practice of providing IVUS support for LMCA PCI when available.

## OCT for LMCA Stenosis

The novel intracoronary imaging modality of OCT provides unique detail of superficial intravascular structures, reviewed in detail elsewhere (9) and has seen important recent advances in the assessment of the LMCA, particularly post-PCI. OCT provides precise information regarding superficial structures down to depths of 2–3 mm within the vessel wall, with axial and lateral resolutions of 20–40 μm, respectively (9). While this allows precise identification of the endothelial surface, and hence, MLA, deeper structures to calculate plaque burden may not be seen (Figure 2). It might be expected that information regarding stenosis severity acquired using OCT is similar to that gained by IVUS; however, a recent comparison suggested that measurements are increased by 8–10% measured by IVUS compared to OCT (10). Long-term outcome data of interventions based on OCT measurements of LMCA stenosis severity are not available, though would seem intuitive, with this caveat.

Optical coherence tomography for assessment of LMCA PCI would appear superior to IVUS, both from the perspective of acute assessment of stent strut apposition (11) and from the longer-term follow-up. The improved axial and lateral resolution is of a magnitude greater with OCT, compared to IVUS, meaning even minor degrees of stent strut mal-apposition are apparent with OCT (11). While improving mal-apposition using OCT would seem intuitively beneficial in improving outcomes post-LMCA stenting, importantly this has not yet been tested in a trial setting, and not yet shown to be associated with improved outcomes.

## Conclusion

Intravascular ultrasound, and more recently OCT, provide important information guiding LMCA assessment both pre- and post-PCI. Given the likely increasing frequency of LMCA intervention, the use of both of these imaging modalities – particularly OCT – is likely to increase. This is an important area of change in the field of intracoronary imaging.

## Conflict of Interest Statement

The author declares that the research was conducted in the absence of any commercial or financial relationships that could be construed as a potential conflict of interest.
